# Crystal structure of bis(μ_2_-4-bromo-2-[({2-[({2-[(5-bromo-2-oxidobenzylidene)amino]ethyl}sulfanyl)sulfonyl]ethyl}imino)methyl]phenolato)dicopper(II) dimethylformamide disolvate

**DOI:** 10.1107/S2056989017015584

**Published:** 2017-10-31

**Authors:** Julia A. Rusanova, Dmytro Bederak

**Affiliations:** a Department of Chemistry, Taras Shevchenko National University of Kyiv, 64/13, Volodymyrska Street, Kyiv, 01601, Ukraine

**Keywords:** crystal structure, dinuclear copper(II) complex, Schiff base, 5-bromo­salicyl­aldehyde, cyste­amine (2-amino­ethanthiol)

## Abstract

The title dinuclear copper(II) complex [Cu_2_(C_18_H_16_Br_2_N_2_O_4_S_2_)_2_] was prepared by direct synthesis of a dianionic Schiff base derived from 5-bromo­salicyl­aldehyde and cyste­amine. The discrete mol­ecules lie across inversion centers and crystallize with two di­methyl­formamide (DMF) mol­ecules of crystallization. The unique Cu^II^ ion is four-coordinated by two tetra­dentate Schiff base ligands in a distorted square-planar N_2_O_2_ environment. In the crystal, short inter­molecular S⋯Br contacts, weak C—H⋯O hydrogen bonds and intra- and inter­molecular π–π stacking inter­actions between rings of the ligand link the components into a two-dimensional network parallel to (010).

## Abstract 2

The crystal structure of the dinuclear copper(II) complex with dianionic Schiff base derived from 5-bromo­salicylic aldehyde and cyste­amine prepared by direct synthesis is reported.

## Chemical context   

Schiff bases and their metal complexes have been studied extensively over the past few decades and represent one of the most widely used organic compounds due to their synthetic flexibility and wide range of applications (Mitra *et al.*,1997 ▸; Bera *et al.*,1998 ▸; Prabhakaran *et al.*, 2004 ▸). Spontaneous self-assembly of Schiff base ligands appears to be an extremely powerful tool for the construction of novel polynuclear compounds. Such complexes having sulfur-containing ligands are of considerable inter­est because of their diverse coordination modes and bridging ability. The formation and cleavage of di­sulfide bonds are known to be important for the biological activity of several sulfur-containing peptides and proteins (Gilbert *et al.*,1999 ▸; Jacob *et al.*, 2003 ▸). It has been shown earlier that copper(II) complexes containing ligands having thio­alkyl moieties are efficient DNA-cleaving agents on treatment with either a reducing agent or on photo-irradiation (Dhar *et al.*, 2005 ▸). In these studies, we continued our investigations in the field of direct synthesis – an efficient method to obtain novel mixed-valence (Kovbasyuk *et al.*, 1997 ▸) and heterometallic complexes with polynuclear (Vassilyeva *et al.*, 1997 ▸; Kovbasyuk *et al.*, 1998 ▸; Semenaka *et al.*, 2010 ▸) and polymeric [Nesterova (Pryma) *et al.*, 2004 ▸, Nesterova *et al.*, 2005 ▸, 2008 ▸] structures. The conditions of direct synthesis influence the spontaneous self-assembly process enabling preparation of coordination compounds with commonly simple ligands *e.g*. amino­alcohols (Vassilyeva *et al.*, 1997 ▸; Kovbasyuk *et al.*,1998 ▸; Semenaka *et al.*, 2010 ▸), ethyl­enedi­amine or related compounds [Kokozay & Sienkiewicz, 1995 ▸; Nesterova (Pryma) *et al.*, 2004 ▸, Nesterova *et al.*, 2005 ▸, 2008 ▸]. The title compound was isolated in an attempt to prepare a heterometallic Cu/Mn complex with a Schiff base ligand, a product of condensation between 5-bromsalicyl­aldehyde and cyste­amine, formed *in situ* in a methanol/di­methyl­formamide (DMF) mixture starting from zero-valent Cu and MnCl_2_. We were unable to obtain the heterometallic complex, nevertheless we suppose that in this system MnCl_2_ catalysed conversion of di­sulfides to thio­sulfonates. Synthesis from the same starting materials with the same conditions without MnCl_2_ leads to a Cu^II^ complex whose structure is very similar to that of the already published compound (CSD refcode FEDCIB; Dhar *et al.*, 2005 ▸).
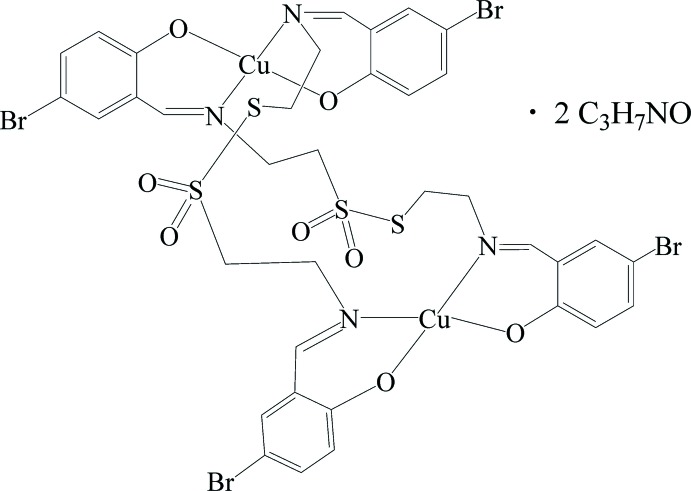


## Structural commentary   

**Figure 1:**
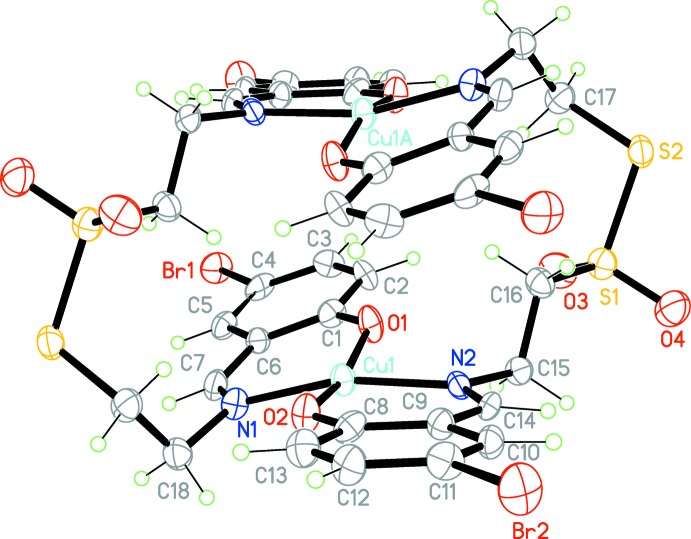
The mol­ecular structure of the title compound with ellipsoids drawn at the 50% probability level. Cu1*A* and unlabelled atoms are generated by the symmetry code (−*x* + 1, −*y* + 2, −*z* + 1). For the sake of clarity, the DMF solvent mol­ecules are not shown.

The title compound is a discrete dinuclear complex that lies across an inversion center (Fig. 1 ▸). The formula unit also contains two DMF mol­ecules of crystallization. The Schiff base acts as a tetra­dentate bridging ligand with each Cu^II^ ion bonded to four donor sites of the ligand. Each Cu^II^ ion in the complex has a distorted square-planar CuN_2_O_2_ environment. The ligand fragments coordinated to Cu^II^ ions are twisted, as defined by the dihedral angle of 22.6 (2)° between the mean planes of atoms O1/N1/C1/C7 and O2/N2/C8/C14. The thio­sulfonate moiety is not involved in a metal–ligand inter­action. The coordination geometry around the Cu^II^ ion is comparable to that found in the aforementioned Cu^II^ complex with a very similar ligand that results from the condensation between salicyl­aldehyde and cyste­amine hydro­chloride (CSD refcode FEDCIB; Dhar *et al.*, 2005 ▸). The separation between the two symmetry-related Cu^II^ ions in the title complex is 4.6533 (15) Å. In general, all bonding parameters and the dimensions of the angles in the title complex are in good agreement with those encountered in related complexes (Dhar *et al.*, 2005 ▸; Zhang *et al.*, 2010 ▸). A fairly short intra­molecular C—H⋯O hydrogen bond is observed (Table 1 ▸).

## Supra­molecular features   

**Figure 2:**
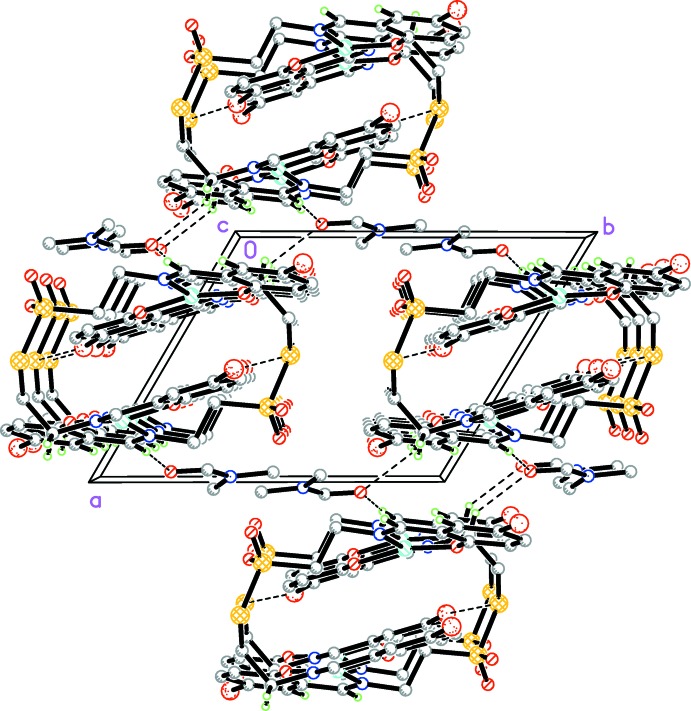
The crystal packing of the title compound viewed along the *c* axis. Short S⋯Br contacts and weak C–H⋯O hydrogen bonds are shown as dashed lines. Only selected H atoms are shown.

**Figure 3:**
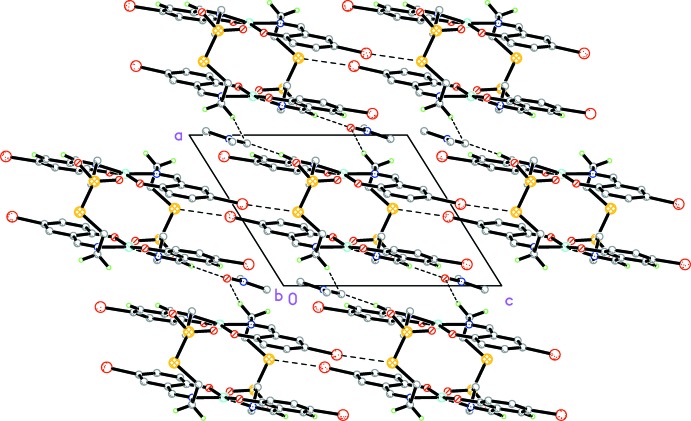
The crystal packing of the title compound viewed along the *b* axis. Short S⋯Br contacts and weak C–H⋯O hydrogen bonds are shown as dashed lines. Only selected H atoms are shown.

In the crystal, weak C—H⋯O hydrogen bonds (Table 1 ▸) connect the solvent DMF mol­ecules to the complex mol­ecules. In addition, short S⋯Br(*x*, *y*, −1 + *x*) contacts [3.4551 (18) Å] connect the complex mol­ecules into chains along [001] (Fig. 2 ▸). Furthermore, π–π stacking inter­actions with a centroid–centroid distance of 3.513 (4) Å for *Cg*1⋯*Cg*3(−*x*, −*y* + 2, −*z* + 1) connect the chains into a two-dimensional network parallel to (010) (Fig. 3 ▸). There is an intra­molecular π–π stacking inter­action between the symmetry-related parts of the complex with a centroid–centroid distance of 3.774 (3) Å for *Cg*1⋯*Cg*2(−*x* + 1, −*y* + 2, −*z* + 1). *Cg*1, *Cg*2 and *Cg*3 are the centroids of the Cu1/O2/C8/C9/C14/N2, C1–C6 and C8–C13 rings, respectively.

## Database survey   

A search of the Cambridge Structural Database (Version 5.38; last update November 2016; Groom *et al.*, 2016 ▸) for related complexes with an amino­ethane­thiol group gave 165 hits, including two closely related structures {bis­[(μ^2^-sulfato)(6-salicyl­idene­amino-3,4-di­thia­hexyl­ammonium)­copper(II)] and bis­[μ^2^-*N*,*N*′-(3,4-di­thia­hexane-1,6-di­yl)bis­(salicylideneimin­ato)-*N*,*N*′,*O*,*O*′]dicopper(II)} with a di­sulfide moiety (Dhar *et al.*, 2004 ▸, 2005 ▸) and similar weak inter­molecular π–π stacking inter­actions (Dhar *et al.*, 2005 ▸). The value of the the S⋯Br contact in the title compound is in good agreement with those in related complexes (CSD refcodes WEMCAT and QELVIN; Salivon *et al.*, 2006 ▸, 2007 ▸; CSD refcode PODDAO; Xia *et al.*, 2008 ▸)

## Synthesis and crystallization   

A solution of KOH (0.12 g, 2 mmol) in minimum amount of methanol was added to a solution of amino­ethane­thiol hydro­chloride (0.23g, 2 mmol) in methanol (5 ml) and stirred in an ice bath for 10 min. The white precipitate of solid KCl was removed by filtration and 5-bromo­salicyl­aldehyde (0.402 g, 2 mmol) in di­methyl­formamide (10 ml) were added to the filtrate and stirred magnetically for 40 min. Copper powder (0.064 g, 1 mmol) and MnCl_2_·4H_2_O (0.198 g, 1 mmol) were added to the yellow solution of the Schiff base formed *in situ*, and the resulting deep green–brown solution was stirred magnetically and heated in air at 323–333 K for 2 h, resulting in a deep-brown precipitate. Crystals suitable for crystallographic study were grown from a saturated solution in DMF after successive addition of CH_2_Cl_2_. The crystals were filtered off, washed with dry *i*-PrOH and finally dried at room temperature (yield: 18%). The IR spectrum of the title compound (as KBr pellets) is consistent with the structural data. It shows all the characteristic functional group peaks in the range 4000–400 cm^−1^: ν(CH) due to aromatic =C—H stretching at 3000–3100 cm^−1^, the aromatic ring vibrations in the 1600–1400 cm^−1^ region, weak S—S absorptions at 500–540 cm^−1^ as well as absorbance at 1630 cm^−1^ assigned to the azomethine ν(C=N) group and ν(SO) at 1330cm^−1^. Analysis calculated for C_42_H_46_Br_4_Cu_2_N_6_O_10_S_4_: C 36.83, H 3.38, N 6.14, S 9.36%; found: C 37.1, H 3.4, N 6.0, S 9.4%.

## Refinement   

Crystal data, data collection and structure refinement details are summarized in Table 2 ▸. All hydrogen atoms were placed in calculated positions and refined in a riding-model approximation.: C—H = 0.95–0.99 Å with *U*_iso_(H) = 1.5*U*_eq_(C-meth­yl) and = 1.2*U*_eq_(C) for other H atoms.

## Supplementary Material

Crystal structure: contains datablock(s) I. DOI: 10.1107/S2056989017015584/lh5856sup1.cif

Structure factors: contains datablock(s) I. DOI: 10.1107/S2056989017015584/lh5856Isup2.hkl

CCDC reference: 1582118

Additional supporting information:  crystallographic information; 3D view; checkCIF report

## Figures and Tables

**Table 1:** Hydrogen-bond geometry (Å, °)

*D*—H⋯*A*	*D*—H	H⋯*A*	*D*⋯*A*	*D*—H⋯*A*
C14—H14⋯O5	0.95	2.32	3.257 (7)	167
C18—H18*A*⋯O5^i^	0.99	2.39	3.201 (7)	138

Symmetry code: (i) 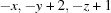.

**Table 2:** Experimental details

Crystal data	
Chemical formula	[Cu_2_(C_18_H_16_Br_2_N_2_O_4_S_2_)_2_]·2C_3_H_7_NO
*M*_r_	1369.81
Crystal system, space group	Triclinic, *P* 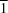
Temperature (K)	173
*a*, *b*, *c* (Å)	10.9140 (4), 12.1104 (5), 12.2394 (5)
α, β, γ (°)	95.620 (2), 116.098 (2), 114.545 (2)
*V* (Å^3^)	1241.05 (9)
*Z*	1
Radiation type	Mo *K*α
μ (mm^−1^)	4.31
Crystal size (mm)	0.25 × 0.12 × 0.04

Data collection	
Diffractometer	Bruker SMART APEXII
Absorption correction	Multi-scan (*SADABS*; Bruker, 2008 ▸)
*T*_min_, *T*_max_	0.68, 0.85
No. of measured, independent and observed [*I* > 2σ(*I*)] reflections	16963, 6224, 3363
*R*_int_	0.094
(sin θ/λ)_max_ (Å^−1^)	0.671

Refinement	
*R*[*F* ^2^ > 2σ(*F* ^2^)], *wR*(*F* ^2^), *S*	0.059, 0.135, 0.97
No. of reflections	6224
No. of parameters	309
H-atom treatment	H-atom parameters constrained
Δρ_max_, Δρ_min_ (e Å^−3^)	0.68, −0.77

Computer programs: *SMART* and *SAINT* (Bruker, 2008 ▸), *SHELXT* (Sheldrick, 2015*a* ▸), *SHELXL2016/4* (Sheldrick, 2015*b* ▸), *SHELXTL* (Sheldrick, 2008 ▸) and *publCIF* (Westrip, 2010 ▸).

## supplementary crystallographic information

### Crystal data

**Table:**

[Cu_2_(C_18_H_16_Br_2_N_2_O_4_S_2_)_2_]·2C_3_H_7_NO	*Z* = 1
*M**_r_* = 1369.81	*F*(000) = 682
Triclinic, *P*1	*D*_x_ = 1.833 Mg m^−^^3^
*a* = 10.9140 (4) Å	Mo *K*α radiation, λ = 0.71073 Å
*b* = 12.1104 (5) Å	Cell parameters from 1470 reflections
*c* = 12.2394 (5) Å	θ = 2.3–20.6°
α = 95.620 (2)°	µ = 4.31 mm^−^^1^
β = 116.098 (2)°	*T* = 173 K
γ = 114.545 (2)°	Plate, brown
*V* = 1241.05 (9) Å^3^	0.25 × 0.12 × 0.04 mm

### Data collection

**Table:**

Bruker SMART APEXII diffractometer	6224 independent reflections
Radiation source: sealed tube	3363 reflections with *I* > 2σ(*I*)
Graphite monochromator	*R*_int_ = 0.094
φ and ω scans	θ_max_ = 28.5°, θ_min_ = 2.0°
Absorption correction: multi-scan (SADABS; Bruker, 2008)	*h* = −14→14
*T*_min_ = 0.68, *T*_max_ = 0.85	*k* = −16→16
16963 measured reflections	*l* = −16→15

### Refinement

**Table:**

Refinement on *F*^2^	0 restraints
Least-squares matrix: full	Hydrogen site location: inferred from neighbouring sites
*R*[*F*^2^ > 2σ(*F*^2^)] = 0.059	H-atom parameters constrained
*wR*(*F*^2^) = 0.135	*w* = 1/[σ^2^(*F*_o_^2^) + (0.032*P*)^2^] where *P* = (*F*_o_^2^ + 2*F*_c_^2^)/3
*S* = 0.97	(Δ/σ)_max_ = 0.006
6224 reflections	Δρ_max_ = 0.68 e Å^−^^3^
309 parameters	Δρ_min_ = −0.77 e Å^−^^3^

### Special details

**Table:**

Geometry. All esds (except the esd in the dihedral angle between two l.s. planes) are estimated using the full covariance matrix. The cell esds are taken into account individually in the estimation of esds in distances, angles and torsion angles; correlations between esds in cell parameters are only used when they are defined by crystal symmetry. An approximate (isotropic) treatment of cell esds is used for estimating esds involving l.s. planes.

### Fractional atomic coordinates and isotropic or equivalent isotropic displacement parameters (Å^2^)

**Table:**

	*x*	*y*	*z*	*U*_iso_*/*U*_eq_	
BR1	0.45806 (7)	0.76624 (6)	−0.04584 (6)	0.03464 (19)	
BR2	0.14178 (8)	1.24956 (7)	0.90146 (7)	0.0395 (2)	
CU1	0.25760 (8)	0.99181 (7)	0.40372 (7)	0.02356 (19)	
S1	0.30738 (18)	0.61966 (14)	0.56704 (15)	0.0279 (4)	
S2	0.51135 (18)	0.64464 (15)	0.72196 (16)	0.0301 (4)	
O1	0.3216 (5)	0.8784 (4)	0.3645 (4)	0.0286 (10)	
O2	0.2484 (5)	1.1322 (4)	0.4727 (4)	0.0298 (10)	
O3	0.2979 (5)	0.5785 (4)	0.4494 (4)	0.0412 (12)	
O4	0.1851 (5)	0.5389 (4)	0.5884 (4)	0.0423 (12)	
O5	0.0481 (6)	0.7837 (5)	0.7614 (5)	0.0486 (13)	
N1	0.2568 (5)	1.0581 (4)	0.2600 (4)	0.0231 (11)	
N2	0.1960 (5)	0.8973 (4)	0.5109 (4)	0.0206 (11)	
N3	0.0268 (6)	0.6113 (5)	0.8280 (5)	0.0268 (12)	
C1	0.3470 (6)	0.8562 (5)	0.2717 (5)	0.0241 (14)	
C2	0.3903 (6)	0.7617 (5)	0.2610 (5)	0.0250 (14)	
H2	0.398268	0.715302	0.319666	0.030*	
C3	0.4202 (7)	0.7372 (6)	0.1676 (6)	0.0291 (15)	
H3	0.449161	0.673889	0.162259	0.035*	
C4	0.4097 (7)	0.8017 (6)	0.0809 (5)	0.0270 (15)	
C5	0.3633 (6)	0.8916 (5)	0.0840 (5)	0.0255 (14)	
H5	0.352938	0.934001	0.021899	0.031*	
C6	0.3312 (6)	0.9203 (5)	0.1798 (5)	0.0203 (13)	
C7	0.2823 (6)	1.0132 (5)	0.1779 (6)	0.0278 (14)	
H7	0.267112	1.045352	0.108075	0.033*	
C8	0.2247 (7)	1.1539 (6)	0.5672 (6)	0.0255 (14)	
C9	0.1854 (6)	1.0633 (5)	0.6268 (5)	0.0216 (13)	
C10	0.1596 (6)	1.0929 (5)	0.7270 (5)	0.0227 (13)	
H10	0.130767	1.031398	0.766808	0.027*	
C11	0.1767 (7)	1.2110 (6)	0.7658 (6)	0.0279 (15)	
C12	0.2151 (7)	1.3013 (5)	0.7078 (6)	0.0277 (14)	
H12	0.225837	1.382493	0.736110	0.033*	
C13	0.2379 (7)	1.2733 (6)	0.6090 (6)	0.0298 (15)	
H13	0.262727	1.335049	0.568479	0.036*	
C14	0.1674 (6)	0.9392 (5)	0.5925 (5)	0.0200 (13)	
H14	0.130912	0.882246	0.633566	0.024*	
C15	0.1701 (6)	0.7651 (5)	0.4940 (5)	0.0220 (13)	
H15A	0.135156	0.722038	0.404124	0.026*	
H15B	0.086443	0.713221	0.510496	0.026*	
C16	0.3263 (7)	0.7752 (5)	0.5895 (5)	0.0261 (14)	
H16A	0.357001	0.813545	0.679209	0.031*	
H16B	0.411204	0.832782	0.576874	0.031*	
C17	0.6560 (7)	0.7086 (5)	0.6740 (6)	0.0287 (15)	
H17A	0.700749	0.652070	0.675639	0.034*	
H17B	0.601620	0.707533	0.584097	0.034*	
C18	0.2096 (7)	1.1545 (6)	0.2377 (6)	0.0297 (15)	
H18A	0.120633	1.133532	0.251267	0.036*	
H18B	0.170671	1.149670	0.146472	0.036*	
C21	0.0446 (7)	0.5001 (5)	0.8217 (6)	0.0374 (17)	
H21A	−0.055679	0.423200	0.796506	0.056*	
H21B	0.128827	0.513038	0.906755	0.056*	
H21C	0.072770	0.488293	0.757429	0.056*	
C23	0.0558 (7)	0.6851 (6)	0.7593 (6)	0.0289 (15)	
H23	0.085171	0.659635	0.703306	0.035*	
C24	−0.0258 (7)	0.6383 (6)	0.9115 (6)	0.0345 (16)	
H24A	0.043606	0.642601	0.998719	0.052*	
H24B	−0.135278	0.569401	0.877022	0.052*	
H24C	−0.021486	0.721154	0.915536	0.052*	

### Atomic displacement parameters (Å^2^)

**Table:**

	*U*^11^	*U*^22^	*U*^33^	*U*^12^	*U*^13^	*U*^23^
BR1	0.0377 (4)	0.0455 (4)	0.0301 (4)	0.0223 (3)	0.0243 (3)	0.0116 (3)
BR2	0.0575 (5)	0.0446 (4)	0.0420 (4)	0.0336 (4)	0.0378 (4)	0.0178 (3)
CU1	0.0301 (4)	0.0239 (4)	0.0229 (4)	0.0149 (3)	0.0173 (3)	0.0104 (3)
S1	0.0320 (9)	0.0245 (8)	0.0316 (9)	0.0159 (7)	0.0186 (8)	0.0102 (7)
S2	0.0337 (9)	0.0331 (9)	0.0356 (9)	0.0204 (8)	0.0225 (8)	0.0212 (8)
O1	0.046 (3)	0.032 (2)	0.027 (2)	0.025 (2)	0.027 (2)	0.018 (2)
O2	0.043 (3)	0.026 (2)	0.033 (2)	0.020 (2)	0.027 (2)	0.014 (2)
O3	0.048 (3)	0.043 (3)	0.041 (3)	0.030 (2)	0.024 (2)	0.013 (2)
O4	0.039 (3)	0.032 (3)	0.063 (3)	0.019 (2)	0.029 (3)	0.023 (2)
O5	0.071 (3)	0.058 (3)	0.075 (4)	0.052 (3)	0.058 (3)	0.053 (3)
N1	0.029 (3)	0.022 (3)	0.023 (3)	0.012 (2)	0.018 (2)	0.007 (2)
N2	0.023 (3)	0.022 (3)	0.020 (2)	0.013 (2)	0.011 (2)	0.012 (2)
N3	0.032 (3)	0.025 (3)	0.029 (3)	0.015 (2)	0.019 (2)	0.011 (2)
C1	0.024 (3)	0.026 (3)	0.022 (3)	0.011 (3)	0.014 (3)	0.005 (3)
C2	0.034 (3)	0.025 (3)	0.022 (3)	0.020 (3)	0.015 (3)	0.012 (3)
C3	0.030 (3)	0.034 (4)	0.026 (3)	0.019 (3)	0.015 (3)	0.007 (3)
C4	0.025 (3)	0.039 (4)	0.020 (3)	0.012 (3)	0.019 (3)	0.006 (3)
C5	0.026 (3)	0.026 (3)	0.023 (3)	0.010 (3)	0.015 (3)	0.008 (3)
C6	0.021 (3)	0.023 (3)	0.018 (3)	0.011 (3)	0.012 (3)	0.008 (3)
C7	0.027 (3)	0.027 (3)	0.029 (3)	0.010 (3)	0.017 (3)	0.014 (3)
C8	0.025 (3)	0.027 (3)	0.028 (3)	0.012 (3)	0.018 (3)	0.009 (3)
C9	0.023 (3)	0.019 (3)	0.025 (3)	0.011 (3)	0.014 (3)	0.008 (3)
C10	0.024 (3)	0.023 (3)	0.023 (3)	0.011 (3)	0.015 (3)	0.011 (3)
C11	0.033 (3)	0.034 (4)	0.024 (3)	0.019 (3)	0.018 (3)	0.007 (3)
C12	0.036 (4)	0.018 (3)	0.032 (4)	0.015 (3)	0.019 (3)	0.008 (3)
C13	0.035 (4)	0.025 (3)	0.029 (3)	0.015 (3)	0.017 (3)	0.007 (3)
C14	0.019 (3)	0.024 (3)	0.023 (3)	0.010 (3)	0.015 (3)	0.012 (3)
C15	0.023 (3)	0.014 (3)	0.030 (3)	0.010 (3)	0.015 (3)	0.008 (3)
C16	0.030 (3)	0.023 (3)	0.025 (3)	0.015 (3)	0.014 (3)	0.010 (3)
C17	0.032 (3)	0.024 (3)	0.037 (4)	0.016 (3)	0.021 (3)	0.012 (3)
C18	0.034 (4)	0.036 (4)	0.033 (4)	0.023 (3)	0.022 (3)	0.019 (3)
C21	0.046 (4)	0.020 (3)	0.043 (4)	0.014 (3)	0.024 (4)	0.010 (3)
C23	0.033 (4)	0.039 (4)	0.033 (4)	0.023 (3)	0.025 (3)	0.019 (3)
C24	0.040 (4)	0.046 (4)	0.034 (4)	0.028 (3)	0.026 (3)	0.012 (3)

### Geometric parameters (Å, º)

**Table:**

BR1—C4	1.910 (5)	C7—H7	0.9500
BR2—C11	1.916 (6)	C8—C9	1.400 (8)
CU1—O2	1.883 (4)	C8—C13	1.411 (7)
CU1—O1	1.888 (4)	C9—C10	1.419 (7)
CU1—N2	1.979 (5)	C9—C14	1.424 (7)
CU1—N1	2.002 (5)	C10—C11	1.369 (8)
S1—O3	1.421 (4)	C10—H10	0.9500
S1—O4	1.442 (4)	C11—C12	1.381 (8)
S1—C16	1.786 (6)	C12—C13	1.376 (8)
S1—S2	2.063 (2)	C12—H12	0.9500
S2—C17	1.823 (6)	C13—H13	0.9500
O1—C1	1.311 (6)	C14—H14	0.9500
O2—C8	1.317 (6)	C15—C16	1.528 (7)
O5—C23	1.229 (7)	C15—H15A	0.9900
N1—C7	1.284 (7)	C15—H15B	0.9900
N1—C18	1.463 (7)	C16—H16A	0.9900
N2—C14	1.288 (6)	C16—H16B	0.9900
N2—C15	1.481 (6)	C17—C18^i^	1.517 (7)
N3—C23	1.324 (7)	C17—H17A	0.9900
N3—C21	1.438 (7)	C17—H17B	0.9900
N3—C24	1.445 (7)	C18—C17^i^	1.517 (7)
C1—C2	1.423 (8)	C18—H18A	0.9900
C1—C6	1.418 (8)	C18—H18B	0.9900
C2—C3	1.358 (7)	C21—H21A	0.9800
C2—H2	0.9500	C21—H21B	0.9800
C3—C4	1.371 (9)	C21—H21C	0.9800
C3—H3	0.9500	C23—H23	0.9500
C4—C5	1.382 (8)	C24—H24A	0.9800
C5—C6	1.415 (7)	C24—H24B	0.9800
C5—H5	0.9500	C24—H24C	0.9800
C6—C7	1.429 (8)		
			
O2—CU1—O1	165.71 (17)	C9—C10—H10	120.3
O2—CU1—N2	92.47 (18)	C10—C11—C12	121.6 (5)
O1—CU1—N2	90.01 (18)	C10—C11—BR2	118.8 (5)
O2—CU1—N1	88.89 (18)	C12—C11—BR2	119.6 (4)
O1—CU1—N1	92.47 (17)	C13—C12—C11	119.8 (6)
N2—CU1—N1	164.48 (18)	C13—C12—H12	120.1
O3—S1—O4	120.1 (3)	C11—C12—H12	120.1
O3—S1—C16	108.6 (3)	C12—C13—C8	120.8 (6)
O4—S1—C16	108.1 (3)	C12—C13—H13	119.6
O3—S1—S2	110.1 (2)	C8—C13—H13	119.6
O4—S1—S2	103.0 (2)	N2—C14—C9	125.9 (5)
C16—S1—S2	106.0 (2)	N2—C14—H14	117.0
C17—S2—S1	103.1 (2)	C9—C14—H14	117.0
C1—O1—CU1	129.9 (4)	N2—C15—C16	108.4 (4)
C8—O2—CU1	129.4 (4)	N2—C15—H15A	110.0
C7—N1—C18	116.9 (5)	C16—C15—H15A	110.0
C7—N1—CU1	123.8 (4)	N2—C15—H15B	110.0
C18—N1—CU1	119.0 (4)	C16—C15—H15B	110.0
C14—N2—C15	115.4 (5)	H15A—C15—H15B	108.4
C14—N2—CU1	124.8 (4)	C15—C16—S1	110.9 (4)
C15—N2—CU1	119.7 (3)	C15—C16—H16A	109.5
C23—N3—C21	121.5 (5)	S1—C16—H16A	109.5
C23—N3—C24	121.4 (5)	C15—C16—H16B	109.5
C21—N3—C24	117.1 (5)	S1—C16—H16B	109.5
O1—C1—C2	118.7 (6)	H16A—C16—H16B	108.1
O1—C1—C6	123.2 (5)	C18^i^—C17—S2	112.3 (4)
C2—C1—C6	118.1 (5)	C18^i^—C17—H17A	109.1
C3—C2—C1	120.5 (6)	S2—C17—H17A	109.1
C3—C2—H2	119.7	C18^i^—C17—H17B	109.1
C1—C2—H2	119.7	S2—C17—H17B	109.1
C2—C3—C4	121.6 (6)	H17A—C17—H17B	107.9
C2—C3—H3	119.2	N1—C18—C17^i^	113.1 (5)
C4—C3—H3	119.2	N1—C18—H18A	109.0
C3—C4—C5	120.5 (5)	C17^i^—C18—H18A	109.0
C3—C4—BR1	119.8 (5)	N1—C18—H18B	109.0
C5—C4—BR1	119.8 (5)	C17^i^—C18—H18B	109.0
C4—C5—C6	119.8 (6)	H18A—C18—H18B	107.8
C4—C5—H5	120.1	N3—C21—H21A	109.5
C6—C5—H5	120.1	N3—C21—H21B	109.5
C5—C6—C1	119.4 (5)	H21A—C21—H21B	109.5
C5—C6—C7	117.5 (5)	N3—C21—H21C	109.5
C1—C6—C7	123.1 (5)	H21A—C21—H21C	109.5
N1—C7—C6	127.2 (6)	H21B—C21—H21C	109.5
N1—C7—H7	116.4	O5—C23—N3	125.9 (6)
C6—C7—H7	116.4	O5—C23—H23	117.0
O2—C8—C9	122.9 (5)	N3—C23—H23	117.0
O2—C8—C13	118.3 (6)	N3—C24—H24A	109.5
C9—C8—C13	118.7 (5)	N3—C24—H24B	109.5
C8—C9—C10	119.7 (5)	H24A—C24—H24B	109.5
C8—C9—C14	124.0 (5)	N3—C24—H24C	109.5
C10—C9—C14	116.3 (5)	H24A—C24—H24C	109.5
C11—C10—C9	119.4 (6)	H24B—C24—H24C	109.5
C11—C10—H10	120.3		

Symmetry code: (i) −*x*+1, −*y*+2, −*z*+1.

### Hydrogen-bond geometry (Å, º)

**Table:**

*D*—H···*A*	*D*—H	H···*A*	*D*···*A*	*D*—H···*A*
C14—H14···O5	0.95	2.32	3.257 (7)	167
C18—H18*A*···O5^ii^	0.99	2.39	3.201 (7)	138

Symmetry code: (ii) −*x*, −*y*+2, −*z*+1.

## References

[bb1] Bera, P., Butcher, R. J. & Saha, N. (1998). *Chem. Lett.* **27**, 559–560.

[bb2] Bruker (2008). *APEX2*, *SMART*, *SAINT*, and *SADABS*. Bruker AXS, Inc., Madison, Wisconsin, USA.

[bb3] Dhar, S., Nethaji, M. & Chakravarty, A. R. (2004). *Dalton Trans.* pp. 4180–4184. 10.1039/b414639e 15573170

[bb4] Dhar, S., Nethaji, M. & Chakravarty, A. R. (2005). *Dalton Trans.* pp. 344–348. 10.1039/b413410a 15616724

[bb5] Gilbert, B. C., Silvester, S., Walton, P. H. & Whitwood, A. C. (1999). *J. Chem. Soc. Perkin Trans. 2*, pp. 1891–1895.

[bb6] Groom, C. R., Bruno, I. J., Lightfoot, M. P. & Ward, S. C. (2016). *Acta Cryst.* B**72**, 171–179. 10.1107/S2052520616003954 PMC4822653 27048719

[bb7] Jacob, C., Giles, G. L., Giles, N. M. & Sies, H. (2003). *Angew. Chem. Int. Ed.* **42**, 4742–4758. 10.1002/anie.200300573 14562341

[bb8] Kokozay, V. N. & Sienkiewicz, A. V. (1995). *Polyhedron*, **14**, 1547–1551.

[bb9] Kovbasyuk, L. A., Babich, O. A. & Kokozay, V. N. (1997). *Polyhedron*, **16**, 161–163.

[bb10] Kovbasyuk, L. A., Vassilyeva, O. Yu., Kokozay, V. N., Linert, W., Reedijk, J., Skelton, B. W. & Oliver, A. G. (1998). *J. Chem. Soc. Dalton Trans.* pp. 2735–2738.

[bb11] Mitra, A., Banerjee, T., Roychowdhury, P., Chaudhuri, S., Bera, P. & Saha, N. (1997). *Polyhedron*, **16**, 3735–3742.

[bb12] Nesterova, O. V., Lipetskaya, A. V., Petrusenko, S. R., Kokozay, V. N., Skelton, B. W. & Jezierska, J. (2005). *Polyhedron*, **24**, 1425–1434.

[bb13] Nesterova, O. V., Petrusenko, S. R., Kokozay, V. N., Skelton, B. W., Jezierska, J., Linert, W. & Ozarowski, A. (2008). *Dalton Trans.* pp. 1431–1436. 10.1039/b713252b 18322622

[bb14] Nesterova (Pryma), O. V., Petrusenko, S. R., Kokozay, V. N., Skelton, B. W. & Linert, W. (2004). *Inorg. Chem. Commun.* **7**, 450–454.

[bb15] Prabhakaran, R., Geetha, A., Thilagavathi, M., Karvembu, R., Krishnan, V., Bertagnolli, H. & Natarajan, K. (2004). *J. Inorg. Biochem.* **98**, 2131–2140. 10.1016/j.jinorgbio.2004.09.020 15541503

[bb16] Salivon, N. F., Filinchuk, Y. E. & Olijnyk, V. V. (2006). *Z. Anorg. Allg. Chem.* **632**, 1610–1613.

[bb17] Salivon, N. F., Olijnik, V. V. & Shkurenko, A. A. (2007). *Russ. J. Coord. Chem.* **33**, 908–913.

[bb18] Semenaka, V. V., Nesterova, O. V., Kokozay, V. N., Dyakonenko, V. V., Zubatyuk, R. I., Shishkin, O. V., Boča, R., Jezierska, J. & Ozarowski, A. (2010). *Inorg. Chem.* **49**, 5460–5471. 10.1021/ic1000123 20540565

[bb19] Sheldrick, G. M. (2008). *Acta Cryst.* A**64**, 112–122. 10.1107/S0108767307043930 18156677

[bb20] Sheldrick, G. M. (2015*a*). *Acta Cryst.* A**71**, 3–8.

[bb21] Sheldrick, G. M. (2015*b*). *Acta Cryst.* C**71**, 3–8.

[bb22] Vassilyeva, O. Yu., Kokozay, V. N., Zhukova, N. I. & Kovbasyuk, L. A. (1997). *Polyhedron*, **16**, 263–266.

[bb23] Westrip, S. P. (2010). *J. Appl. Cryst.* **43**, 920–925.

[bb24] Xia, J.-H., Liu, Z. & Jin, L.-X. (2008). *Chin. J. Inorg. Chem.* **5**, 823–826.

[bb25] Zhang, S.-H., Wang, Y., Feng, C. & Li, G. Z. (2010). *J. Coord. Chem.* **63**, 3697–3705.

